# Wafer-Scale Hierarchical Nanopillar Arrays Based on Au Masks and Reactive Ion Etching for Effective 3D SERS Substrate

**DOI:** 10.3390/ma11020239

**Published:** 2018-02-04

**Authors:** Dandan Men, Yingyi Wu, Chu Wang, Junhuai Xiang, Ganlan Yang, Changjun Wan, Honghua Zhang

**Affiliations:** Jiangxi Key Laboratory of Surface Engineering, Jiangxi Science and Technology Normal University, Nanchang 330013, China; mendandan1999@126.com (D.M.); yingyiwu1994@163.com (Y.W.); m17370018946@163.com (C.W.); xiangjunhuai@163.com (J.X.); yangjiangmail@163.com (G.Y.); 18870804638@163.com (C.W.)

**Keywords:** wafer-scale, hierarchical arrays, RIE, Ag NPs, SERS substrate

## Abstract

Two-dimensional (2D) periodic micro/nanostructured arrays as SERS substrates have attracted intense attention due to their excellent uniformity and good stability. In this work, periodic hierarchical SiO_2_ nanopillar arrays decorated with Ag nanoparticles (NPs) with clean surface were prepared on a wafer-scale using monolayer Au NP arrays as masks, followed by reactive ion etching (RIE), depositing Ag layer and annealing. For the prepared SiO_2_ nanopillar arrays decorated with Ag NPs, the size of Ag NPs was tuned from ca. 24 to 126 nanometers by controlling the deposition thickness of Ag film. Importantly, the SiO_2_ nanopillar arrays decorated with Ag NPs could be used as highly sensitive SERS substrate for the detection of 4-aminothiophenol (4-ATP) and rhodamine 6G (R6G) due to the high loading of Ag NPs and a very uniform morphology. With a deposition thickness of Ag layer of 30 nm, the SiO_2_ nanopillar arrays decorated with Ag NPs exhibited the best sensitive SERS activity. The excellent SERS performance of this substrate is mainly attributed to high-density “hotspots” derived from nanogaps between Ag NPs. Furthermore, this strategy might be extended to synthesize other nanostructured arrays with a large area, which are difficult to be prepared only via conventional wet-chemical or physical methods.

## 1. Introduction

Surface enhanced Raman scattering (SERS) has been considered as a promising spectroscopic technique for label-free and nondestructive trace detection for biological and chemical analytes at super low concentration with ultra-sensitivity [1,2,3,4,5,6,7]. The Raman scattering signals of the analytes adsorbed on a rough surface of plasmonic metals could be amplified by several orders of magnitude [8,9,10,11]. Noble metal (Au and Ag) nanoparticles (NPs) can generate intense electromagnetic field due to localized surface plasmon resonance (LSPR), resulting in excellent activity as a SERS substrate [9]. The places where the electromagnetic field is extremely strong for enhancing Raman scattering are called “hotspots”, which mainly depend on the nanogap, morphology, size and nature of the metal materials [12]. In recent decades, significant efforts have been raised to construct SERS substrates by synthesizing Au, Ag and Au–Ag alloyed NPs with different size and morphologies, such as concave trisoctahedral and calyptriform Au nanocrystal [13], porous Au NPs [14], porous Au–Ag alloyed nanocubes [15], Ag–Au hybrid nanosponges [16] and AuAg bimetallic nanoparticles [17]. For example, Gao and co-workers fabricated porous Au–Ag alloy NPs with clean surface via a dealloying process. The high porosity of theses Au–Ag alloyed NPs can produce abundant inherent “hotspots” and the clean surfaces make these “hotspots” readily accessible for target molecules. These conditions make the producing Au–Ag alloyed NPs show excellent SERS activity [18].

The uniformity and reproducibility of the SERS substrate should also be improved in addition to the activity of the SERS substrate. Two-dimensional (2D) periodic micro/nanostructured arrays using as SERS substrates have attracted intense attention due to their excellent uniformity and good stability [19,20,21,22,23]. This kind of SERS substrates was mainly constructed using monolayer colloidal crystals as template, followed by solution-dipping or electrochemical deposition technique [20,21,22,23]. For instance, Meng and co-workers fabricated hierarchical periodic Ag nanorod bundles arrays as SERS substrates with good uniformity and reproducibility. The Ag nanorod bundles arrays were constructed by using 2D ordered anodic aluminum oxide (AAO) membrane and polystyrene (PS) nanosphere array as a dual template, followed by electrodepositing. In each bundle, “hot spots” are generated between adjacent Ag nanorods after solution evaporation, leading to the SERS sensor with high sensitivity [23]. However, although these methods have the advantages of low costs, they need complex and strict preparation procedure. Additionally, these fabrication methods suffer from significantly low production yield as only a small area array is fabricated in each process, which seriously hinders their further practical applications. Over the past decade, many significant efforts have been devoted to maximizing the sensitivity of these substrates rather than addressing the issue of expanding the area. Nevertheless, for practical applications it remains highly desirable to prepare SERS substrates with a large area. 

In this work, a facile method has been presented to fabricate 2D SiO_2_ nanopillars decorated with Ag NPs arrays as SERS substrates in a large area with a well-controllable morphology. The 2D SiO_2_ nanopillar array was prepared using 2D Au NPs arrays as a template combined with reactive ion etching (RIE). After depositing a layer of Ag on the as-prepared SiO_2_ nanopillar array, it was annealed at 600 °C for 2 h to form 2D SiO_2_ nanopillar arrays decorated with Ag NPs. The area of as-obtained arrays can achieve wafer-scale with a very uniform morphology. Importantly, after depositing 30 nm Au layer, the SiO_2_ nanopillar decorated with Ag NPs array could be used as highly sensitive SERS substrates for the detection of 4-aminothiophenol (4-ATP) and rhodamine 6G (R6G). By effectively controlling the deposition thickness of Ag layer, the size of Ag NPs could be tuned from ca. 24 to 126 nm. When the deposition thickness of Ag layer is 30 nm, the SiO_2_ nanopillar arrays decorated with Ag NPs possess highly sensitive SERS activity. The excellent SERS performance of this substrate is mainly attributed to high-density “hotspots” derived from nanogaps between Ag NPs. Furthermore, this strategy might be extended to synthesize other nanostructures with a large area, which are difficult to be prepared only via conventional wet-chemical or physical methods. 

## 2. Materials and Methods 

### 2.1. Materials

Polystyrene (PS) microspheres suspension (2.50 wt. % in water, 500 nm in diameter) was purchased from Alfa Aesar Corporation (Heysham, UK). The reagents including ethanol, acetone, H_2_SO_4_ and NH_3_**^.^**H_2_O were bought from Sinopharm Chemical Reagent Co., Ltd. (Shanghai, China). 4-ATP and R6G were obtained from Aladdin Industrial Corporation (Hangzhou, China). SiO_2_ substrate was offered by TRION Technology Corporation (Tempe, AZ, USA). The targets of pure Au and Ag (99.99%) were bought from ZhongNuo Advanced Material Technology Corporation (Beijing, China). Deionized water was prepared with a Milli-Q water purification system (Merck, Darmstadt, Germany). All the chemicals were of analytical grade and were used without further purification.

### 2.2. Fabrication of the SiO_2_ Nanopillar Arrays Decorated with Ag NPs 

**Figure 1:**
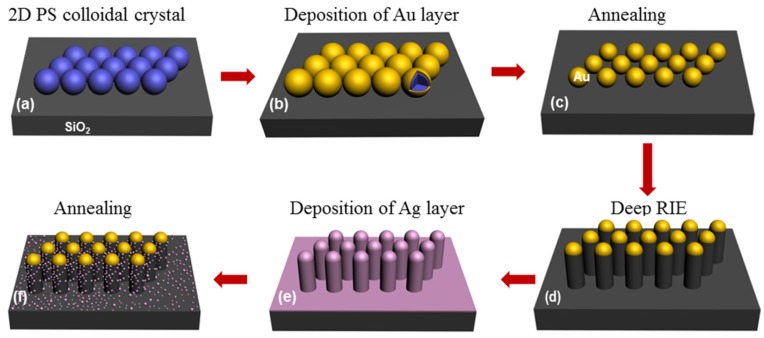
Schematic diagrams of fabrication of the SiO_2_ nanopillar arrays decorated with Ag NPs. (**a**) Monolayer PSs was fabricated on SiO_2_ wafer by self-assembling process at gas-liquid interface; (**b**) A certain thickness of Au film was deposited onto surface of PS spheres by magnetron sputtering method; (**c**) After annealing at 1000 ^o^C for 2 h to remove the template of PSs, and Au NPs array was formed; (**d**) The SiO_2_ nanopillar arrays were fabricated by deep RIE process; (**e**) A layer of Ag film was deposited on the as-formed SiO_2_ nanopillar arrays by an ion-beam coater with a sputtering current of 20 mA under different deposition time; (**f**) After annealing at 600 °C for 2 h, SiO_2_ nanopillar arrays decorated with Ag NPs were formed.

The hierarchical SiO_2_ nanopillar arrays decorated with Ag NPs @ Au film were fabricated as following process (Figure 1). Briefly, 2D Au NPs arrays with hexagonal non-close-packed (HNCP) arrangement on SiO_2_ substrate was fabricated by using the 2D PS colloidal crystal as a template and further annealing, as previously reported (Figure 1a–c) [24,25]. Subsequently, the as-prepared 2D Au NPs arrays on the SiO_2_ substrate with the periodic length of 500 nm and diameter of 260 nm as a mask was put into the RIE chamber (Sirus T2, TRION Technology, Clearwater, FL, USA) for etching (Figure 1d). During the RIE process, etching-reactive gases CHF_3_ and O_2_ were introduced into the chamber with a flow of 46 and 4 sccm, under the pressure at 120 mTorr, etching power of 150 W and etching time for 1500 s, respectively. The SiO_2_ nanopillar arrays were fabricated in this process. After that, a layer of Ag was deposited on the as-formed SiO_2_ nanopillar arrays by an ion-beam coater with a sputtering current of 20 mA under different deposition time. To make the Ag film disperse evenly on the surfaces of the nanopillars, during the depositing process of Ag layer, the samples were placed at 45° for the first deposition and then rotated about 90° along the normal of sample for further depositing with the same parameters until return to original position. The thickness of Ag layer was tuned by deposition time. Then, the SiO_2_ nanopillar arrays with deposited Ag layer was calcined at 600 °C for 2 h to form the SiO_2_ nanopillar arrays decorated with Ag NPs. Finally, 30 nm Au film was deposited on the as-prepared SiO_2_ nanopillar arrays decorated with Ag NPs before further SERS measurement.

### 2.3. Characterization

The morphologies of as-prepared samples were characterized by a field-emission scanning electron microscope (FE-SEM, Sirion200, FEI, Hillsboro, OR, USA). 4-ATP and R6G were used as a probe molecule to evaluate the SERS performance of the as-fabricated active substrates. SERS measurements were conducted by a confocal microprobe Raman spectrometer (Reni Shaw inVia Reflex, London, UK) with a laser beam of 532 nm wavelength. The laser operated at 5 mW and the integral time was 9 s. Before SERS measurements, the as-fabricated active substrates were immersed in 10 mL 4-ATP alcoholic solution or R6G aqueous solution with different concentrations for 30 min, cleaned with deionized water several times, and then dried in a flow of nitrogen. For comparison, all the samples share the same condition of adsorption and measurement of Raman spectrum.

## 3. Results and Discussion

### 3.1. The SiO_2_ Nanopillar Arrays

**Figure 2:**
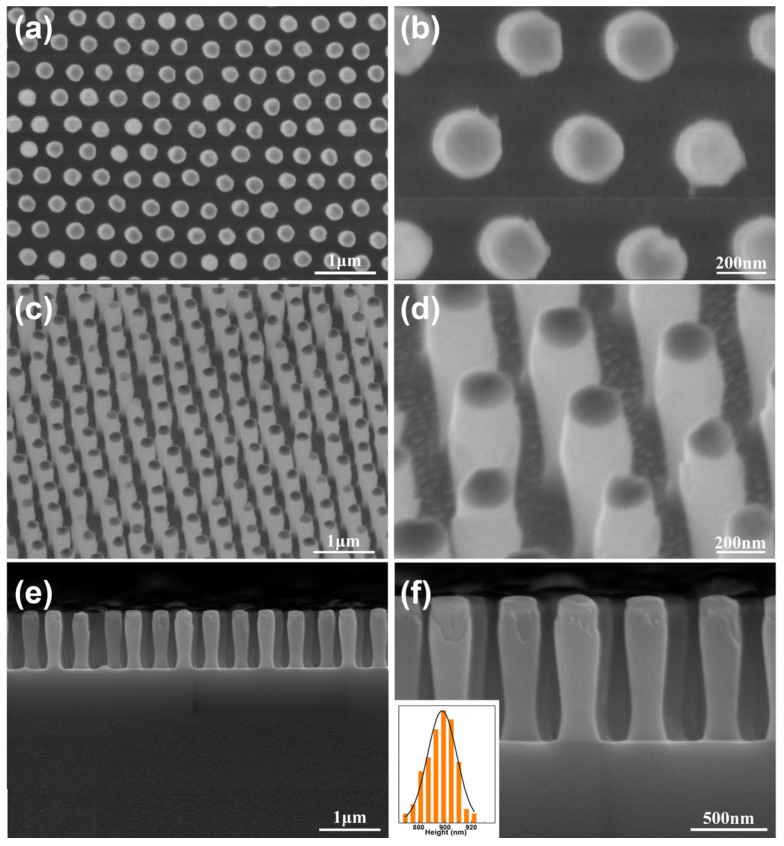
FE-SEM images of periodic SiO_2_ nanopillar arrays fabricated under etching time of 1500 s: (**a**), (**c**) and (**e**) is low-magnification image observed from the top, tilted and cross-sectional view, respectively; (**b**), (**d**) and (**f**) is the corresponding expanded image of (**a**), (**c**) and (**e**) respectively; The inset in (**f**) shows the height distribution of nanopillars in the array.

Ordered SiO_2_ nanopillar arrays were formed under a certain etching power (150 W), etching pressure (120 mTorr) by using monolayer Au NP arrays as masks. Figure 2 displays the FE-SEM images of the SiO_2_ nanopillar arrays under etching time of 1500 s. From the top-view SEM images, it is apparent that all of the nanopillars are arranged in an ordered HNCP alignment with a periodicity of 500 nm (Figure 2a). The diameter of each structural unit is ca. 220 nm (Figure 2b). Each structural unit has a nanopillar-shape (Figure 2c). There is an Au NP on the top of each nanopillar (Figure 2d), resulting from the residue of the mask. The height of the SiO_2_ nanopillar is ca. 898 nm (Figure 2e,f). Additionally, the diameter of lower half of the nanopillar is slightly smaller than that of the upper part, mainly due to the effect of isotropic etching. The longitudinal etching also occurs at different degrees in the etching process. A detailed statement about device and mechanism of RIE was reported in our previous work [19].

### 3.2. The Hierarchical SiO_2_ Nanopillar Decorated with Ag NPs Arrays

**Figure 3:**
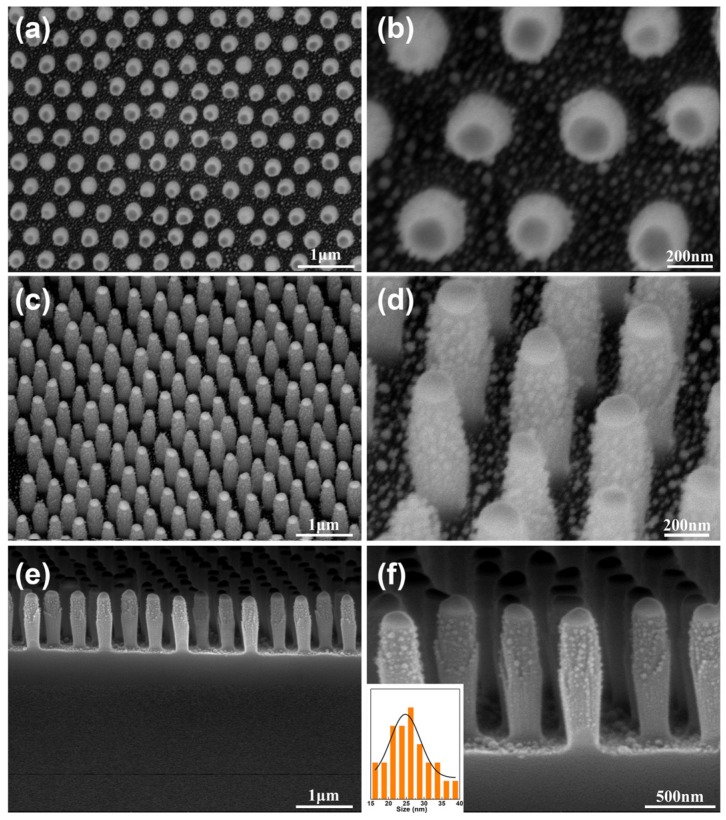
FE-SEM images of periodic SiO_2_ nanopillar decorated with Ag NPs arrays fabricated under deposition thickness of Ag layer of 20 nm: (**a**), (**c**) and (**e**) is low-magnification image observed from the top, tilted and cross-sectional view, respectively; (**b**), (**d**) and (**f**) is the corresponding expanded images of (**a**), (**b**) and (**e**); The inset in (**f**) shows the size distribution of Ag NPs on the SiO_2_ nanopillars.

To decorate SiO_2_ nanopillar array with Ag NPs, an Ag layer with a certain thickness was deposited onto the top and sidewall of the as-prepared SiO_2_ nanopillar arrays by magnetron sputtering deposition technology. The periodic SiO_2_ nanopillar arrays decorated with Ag NPs were obtained by annealing the SiO_2_ nanopillar array with Ag coating in a furnace at 600 °C for 2 h. The morphology of Ag NPs can be tuned by the deposition thickness of the Ag layer. As observed in Figure 3, the Ag NPs were successfully synthesized on the nanopillars. The regularity and interspacing of the array exhibit no significant change, as shown in Figure 3a,b. The distribution of the Ag NPs on the surface of the SiO_2_ nanopillars with a size of ca. 24 nm is disorderly (Figure 3c,d). Additionally, as shown in Figure 3e,f, the bottom region of the nanopillars also contains a large number of Ag NPs, and the Au NPs as a mask were transformed into discal Au NPs. Meanwhile, elemental mapping images of the SiO_2_ nanopillar array decorated with Ag NPs (Supplementary Materials, Figure S1) revealed the existence of Si, O and Ag elements dispersed in the position of nanopillars and the bottom region with SiO_2_ being a majority. The Au element mainly dispersed on the top of nanopillars. Further studies show that Ag element also appeared at the top of nanopillars, indicating that Au–Ag alloy NPs formed. Additionally, in Figure 3f, it can be seen that there are no obvious Ag NPs presenting on the surfaces of the discal Au NPs. This case was attributed to the depositing Ag film in this region being melted with Au NPs to form Au–Ag alloy NPs, which was consistent with elemental mapping image. Moreover, the size of Ag NPs in the upper part of the nanopillar is larger than that in the lower part (Figure 3f). The reason is likely to be that Ag atoms (group) from the multiple directions at the top of nanopillars would be blocked by approaching nanopillars, resulting in a thicker Ag film in upper part of nanopillar than in the bottom half due to the multidirectional deposition and shadow effect in the process of sputtering deposition. 

**Figure 4:**
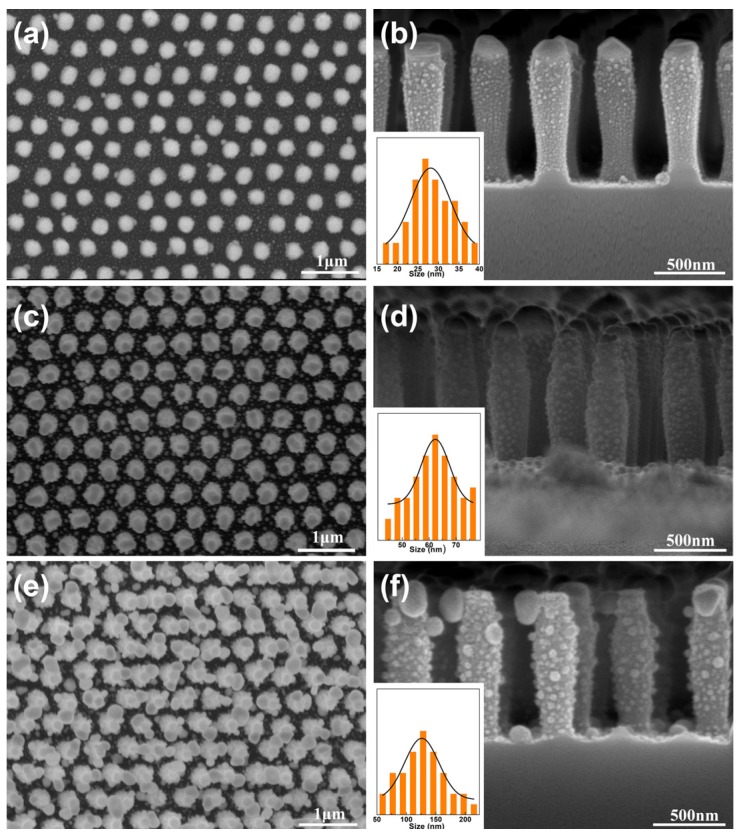
FE-SEM images of periodic SiO_2_ nanopillar arrays decorated with AgNPs fabricated under different deposition thickness of Ag layer. (**a**), (**c**) and (**e**) is low-magnification top view image of deposition thickness of Ag layer of 30 nm, 60 nm and 90 nm, respectively; and (**b**), (**d**) and (**f**) is the corresponding expanded cross-sectional image of (**a**), (**c**) and (**e**), respectively. The insets in (**b**), (**d**) and (**f**) show the corresponding size distribution of Ag NPs on the SiO_2_ nanopillars.

Apart from the above-mentioned deposition thickness of Ag layer with 20 nm, four additional samples with the 30 nm, 60 nm and 90 nm thickness of deposited Ag layer have also been prepared. The morphology of the sample changes gradually with the increase of the thickness of Ag layer. When the thickness increased to 30 nm, the hierarchical SiO_2_ nanopillar arrays decorated with Ag NPs has a similar morphology as that of 20 nm, as shown in Figure 4a,b. If the deposition thickness of Ag layer increased to 60 nm, the size of Ag NPs increased to ca. 62 nm, as shown in Figure 4c,d. However, if the thickness further increased to 90 nm, the morphology of the sample would change greatly, as shown in Figure 4e,f. The periodicity of the structural array was destroyed and the size of the Ag NPs reached up to ca. 126 nm, making the adjacent structural units link together. Subsequently, the hierarchical periodic arrays composed of SiO_2_ nanopillar arrays decorated with Ag NPs with deposition thickness of 20 nm, 30 nm, 60 nm and 90 nm were selected for further SERS investigation. A rough surface is important to increase the SERS signals [21]. To increase the surface roughness, 30 nm Au layer was deposited on the as-prepared SiO_2_ nanopillar arrays decorated with Ag NPs before further SERS measurement. A comparison of Au–Ag alloy NP before and after depositing Au film was studied, as shown in Figure S2 (Supplementary Materials). In Figure S2, it can be seen that the surface became rougher after depositing Au film.

### 3.3. SERS Enhancement of Hierarchical SiO_2_ Nanopillar Arrays Decorated with Ag NPs 

In this work, 4-ATP was used as a molecule to evaluate enhancement of Raman signals of the as-prepared SERS active substrates. Raman spectra of 10^−4^ M 4-ATP on the SiO_2_ nanopillars array, SiO_2_ nanopillars with the Ag film, SiO_2_ nanopillar arrays decorated with Ag NPs and SiO_2_ nanopillar arrays decorated with Ag NPs after depositing Au film are shown in Figure S3 (Supplementary Materials). Compared with the SiO_2_ nanopillars, the Raman signal intensity of SiO_2_ nanopillars with the Ag film was enhanced. This enhancement can be attributed to increase surface roughness arising from depositing Au layer. Furthermore, when the Ag layer deposited on SiO_2_ nanopillars was changed into Ag NPs by annealing treatment, the SERS signals rapid declined, because the surfaces of these Ag NPs became smooth. However, when a layer of Au film was deposited onto the SiO_2_ nanopillar arrays decorated with Ag NPs, the SERS signals was considerably enhanced due to increasing surface roughness. Compared with the SiO_2_ nanopillar arrays with Ag film, the Raman signal intensity (1080 cm^−1^ for 4-ATP) of SiO_2_ nanopillar array decorated with Ag NPs after depositing Au film was considerably enhanced and increased ca. 7 times. This case was mainly attributed to the increasing number of “hotspots” originated from the Ag NPs on the SiO_2_ nanopillars. The as-prepared hierarchical SiO_2_ nanopillar array decorated with Ag NPs @ Au film possess rough surface and abundant SERS “hotspots”, and they could be used as a SERS-active substrate.

**Figure 5:**
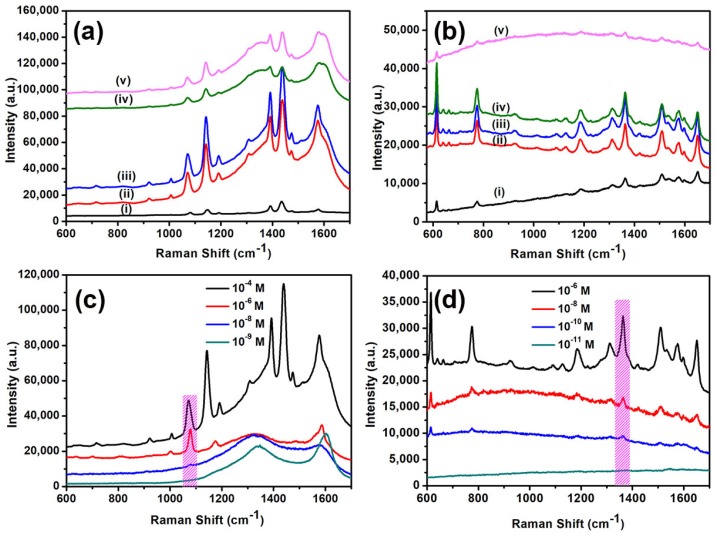
Comparison of average Raman spectra for: 10^−4^ M 4-ATP (**a**); and 10^−^^6^ M R6G (**b**), on the as-prepared periodic hierarchical SiO_2_ nanopillar arrays decorated with Ag NPs after deposition 30 nm Au film and reference sample. The five curves of Raman spectra from bottom to top correspond to: reference sample (i); and the substrates with the deposition Ag layer thickness of: 20 nm (ii); 30 nm (iii); 60 nm (iv); and 90 nm (v). Raman spectra on SiO_2_ nanopillar arrays decorated with Ag NPs after deposition 30 nm Au film with different concentrations of: 10^−^^4^ M to 10^−^^9^ M 4-ATP (**c**); and 10^−^^6^ M to 10^−^^11^ M R6G (**d**).

Raman spectra of the 10^−4^ M 4-ATP on substrates with different morphologies is shown in Figure 5. All samples were immersed in 10^−4^ M 4-ATP solution for 30 min and dried in air before detection. A SiO_2_ wafer with a flat surface-coated 30 nm Au film served as the reference sample and its Raman spectra was observed in Curve (i) of Figure 5a. The other four curves in Figure 5 from bottom to top correspond to Raman spectra of the substrates with different deposition thickness of Ag layer before annealing: 20 nm (ii); 30 nm (iii); 60 nm (iv); and 90 nm (v). As shown in Figure 5a, the obtained hierarchical SiO_2_ nanopillar arrays decorated with Ag NPs after depositing 30 nm Au film reveal obvious SERS enhancements compared with the reference sample. The peak intensity slightly increased with increasing deposition thickness below 30 nm, but decreased when the deposition thickness exceeded 30 nm. Beside 4-ATP, the hierarchical SiO_2_ nanopillar arrays decorated with Ag NPs exhibited good SERS performance for R6G. The similar tendency was observed as 4-ATP when 10^−6^ M R6G was detected under different morphologies, as shown in Figure 5b. Hence, the as-prepared periodic arrays achieved the optimal SERS activity at the deposition Ag layer thickness of 30 nm. 

Generally, SERS enhancement is mainly dependent on the shape, size, nanogaps and aggregation state of structure units [12]. The rough surface is also important to increase the SERS signals [21]. Compared with the SiO_2_ wafer with a flat surface, the surfaces of the obtained SiO_2_ nanopillar decorated with Ag NPs arrays become rougher, resulting in the enhanced SERS signal. Additionally, these Ag NPs decorated on the nanopillar can produce plasmonic coupling under light excitation, resulting in electromagnetic field enhancement and forming a large quantity of efficient “hotspots” [26]. The change in the SERS effect on the periodic arrays with different morphologies originates from the change of size and nanogaps between the Ag NPs. As discussed above, the depositing Ag thickness has an important influence on the morphology of the SiO_2_ nanopillar arrays decorated with Ag NPs. When the deposition thickness was 20 nm, the size of the Ag NPs irregularly presented on SiO_2_ nanopillar arrays was about a dozen. When depositing thickness increased to 30 nm, the size of disordered Ag NPs increased slightly, and nanogaps decreased. Compared with the deposition thickness of 20 nm, the decreased nanogaps result in a slight increase of the SERS signal [27]. However, if the depositing thickness further increased to 60 nm, the size of Ag NPs would increase to tens of nanometers. Furthermore, the corresponding value would reach ca. 126 nm at higher depositing thickness of 90 nm. The intensity of peaks in SERS spectra declined sharply with the increase of the size of Ag NPs. This case was attributed to the decreasing number of “hotspots” and increasing value of nanogaps. Therefore, when the deposition thickness was 30 nm, the nanogaps between the Ag NPs would obtain an appropriate value and a large amount of “hotspots” were generated. As a result, a strong SERS signal was obtained successfully. 

Moreover, the detection limit of 4-ATP and R6G of the as-prepared hierarchical SiO_2_ nanopillar array decorated with Ag NPs (deposition thickness of Ag layer: 30 nm) was studied. Figure 5c demonstrates the intensity of Raman spectra of 4-ATP at different concentrations from 10^−6^ to 10^−9^ M on the SiO_2_ nanopillar arrays decorated with Ag NPs after depositing 30 nm Au film. One can find that the intensity of the SERS signal gradually decreased with a decrease of 4-ATP concentration. At minimal 4-ATP concentration, the SERS peaks could not be identified. Therefore, the detection limit of SiO_2_ nanopillar arrays decorated with Ag NPs could be estimated as 10^−8^ M. The detection limit of R6G was 10^−10^. However, the detection limit is low compared to other SERS substrates, e.g., sponge-like porous Au–Ag alloy nanocubes (10^−10^ M for 4-ATP) [15], hydrogel microsphere @ Au nanospheres Au film (10^−11^ M for 4-ATP) [12], and honeycomb-shaped Au arrays with periodicity of 350 nm (10^−11^ M for R6G) [21], due to lack of sufficient “hotspots”. Therefore, the number of “hotspots” should be further increased.

## 4. Conclusions

The wafer-scale periodic SiO_2_ nanopillar arrays were prepared by using monolayer Au NP arrays as masks, followed by RIE. After decorating with Ag NPs, these periodic hierarchical nanostructured arrays were used as SERS active substrates and presented obvious SERS signals for 4-ATP and R6G. The detection sensitivity of SERS could be tuned by the size of Ag NPs of the SiO_2_ nanopillar arrays decorated with Ag NPs. In contrast to other SERS substrates with different deposition thickness of Ag layer, the SERS substrate with the deposition thickness of 30 nm exhibits the best SERS performance. The excellent SERS performance of this substrate is mainly attributed to high-density “hotspots” derived from nanogaps between Ag NPs. Furthermore, this strategy might be extended to synthesize other nanostructured arrays with a large area, which are difficult to prepare only via conventional wet-chemical or physical methods.

## Supplementary Materials

The following are available online at http://www.mdpi.com/1996-1944/11/2/239/s1, Figure S1: Elemental mapping images of the SiO_2_ nanopillar arrays decorated with Ag NPs; Figure S2: Comparison of surfaces for Au-Ag alloy NP on the as-prepared periodic hierarchical SiO_2_ nanopillar array: before (**a**); and after (**b**) depositing Au film; Figure S3: Comparison of average Raman spectra for the 10^−4^ M 4-ATP on: the SiO_2_ nanopillars array (i); the SiO_2_ nanopillars with the Ag film (ii); the SiO_2_ nanopillar arrays decorated with Ag NPs (iii); and the SiO_2_ nanopillar arrays decorated with Ag NPs after depositing Au film (iv).
