# Supplementary material for: Wafer-Scale Hierarchical Nanopillar Arrays Based on Au Masks and Reactive Ion Etching for Effective 3D SERS Substrate
Source: Materials (Basel). 2018 Feb 4;11(2):239. doi: 10.3390/ma11020239 (PMC5848936; doi:10.3390/ma11020239)
Supplement: Supplementary file 1 [file materials-11-00239-s001.pdf]

Wafer-scale Hierarchical Nanopillar Arrays Based on Au Masks and Reactive Ion Etching for Effective 3D SERS Substrate

Dandan Men, Yingyi Wu, Chu Wang, JunhuaiXiang, Ganlan Yang, Honghua Zhang*

Jiangxi Key Laboratory of Surface Engineering, Jiangxi Science and Technology Normal University, Nanchang, Jiangxi 330013, P. R. China

*Correspondence: zhanghonghua@impcas.ac.cn; Tel.:15270008537

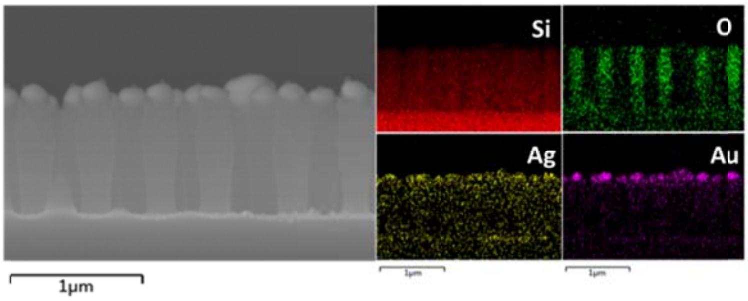

Figure S1. Elemental mapping images of the SiO_2 nanopillar arrays decorated with Ag NPs.

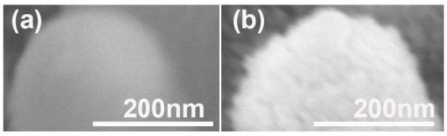

Figure S2. Comparison of surfaces for Au-Ag alloy NP on the as-prepared periodic hierarchical SiO_2 nanopillar array before (a) and after (b) depositing Au film.

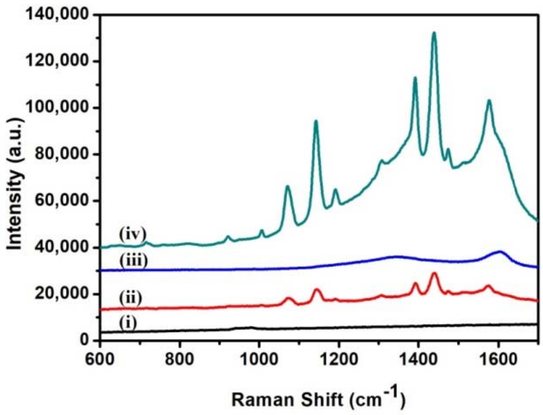

Figure S3. Comparison of average Raman spectra for the 10^{-4} M 4-ATP on the SiO_2 nanopillars array (i), SiO_2 nanopillars with the Ag film (ii), SiO_2 nanopillar arrays decorated with Ag NPs (iii) and SiO_2 nanopillar arrays decorated with Ag NPs after depositing Au film (iv).
